# Unqualified Practice

**Published:** 1915-11-27

**Authors:** 


### The Hospital

The Workers' Newspaper of Administrative Medicine and Institutional Life, Administration, National Insurance and Health.

No. 1537, Vol. LIX. SATURDAY, NOVEMBER 27, 1915. PRICE ONE PENNY.

### UNQUALIFIED PRACTICE.

At the opening of the winter session of the Pharmaceutical Society's School of Pharmacy the distinguished surgeon who delivered the introductory address took occasion to warn his hearers against the impression that their dispensing acquaintance with drugs could fit them in any measure to undertake the treatment of disease. The proposition is, of course, beyond dispute, seeing that the wise and intelligent use of remedies must necessarily be preceded by accurate diagnosis, and in this latter art the pharmacist has neither education nor skill. Hence no medical practitioner can admit, or ought to admit, that any person who has not had an adequate medical training can possibly be regarded as a reliable adviser in a case of illness, whether this is obviously severe or apparently trivial. What seems to the untrained observer a slight and simple matter may be the beginning of serious mischief, which, possibly, on complete recognition and treatment, might be checked at the outset. Thus the sufferer who wishes for the maximum degree of safety must, however slight his suffering, place himself in the care of a duly qualified and registered medical practitioner. This is the professional doctrine and standpoint, and though it may be qualified as a counsel of perfection its prudence and validity can hardly be gainsaid. We must therefore agree that the selection of a pharmacist or other unqualified person as a guide in the treatment of disease is an act which falls short of the highest wisdom. It is to incur a risk which might be avoided.

While accepting these propositions, and even emphasising them, there yet remains the notorious fact that as a guide to conduct they are very largely ignored. The amateur prescriber is legion, and his friendly intentions, if not actually welcomed, are at least rarely resented. Nostrums and cures crowd the advertisement columns of the most respectable newspapers and magazines, and not a few bring handsome incomes to their proprietors. And gentlemen with double-barrelled names and severely personal methods receive compliments on their skill and testimonies to their success from members of even the highest social ranks. In each and all of these transactions there are necessarily two parties concerned, and if censure is to be passed on such proceedings the question may be asked in which direction it should fall. When the supply is so abundant it is a reasonable presumption that the demand is not a scanty one, and it may be that exhortation would be more fruitful were it directed less to the prescriber and more to the public. Agreed it is unwise to solicit the pharmacist or other unqualified person as a medical adviser, yet the first step is taken by the patient, and harm, if harm there be, is reserved for the patient. There would therefore seem to be reasonableness in the suggestion that if a change of habit is to be accomplished it must be sought rather in the education of the public than in the censure of the unqualified adviser. Cut off the demand and the supply will cease automatically. This task is a formidable one, but we doubt whether anything less than its accomplishment will succeed in bringing the administration of medical advice into harmony with the strict principles of prudent conduct. The alternative is to call for the strong arm of the law to put down unqualified practice under severe penalties. Now whoever else may invoke this aid the medical practitioner is hardly in a position to advocate it. In such a capacity he is liable to the taunt which weakens any reformer—namely, that he has a motive of self-interest in the proposal —and to hope for success in such circumstances is a vain thing. Further, as a matter of dignity, it is no part of the policy of the medical profession to promote legal action which would have as its consequence, if not as its motive, the sweeping of all sufferers into the doctor's consulting room. If unqualified practice is to be "put down," the end will not be reached through the instrumentality of the medical profession. The only other force available is the pressure of public opinion, which brings us back to the point of a moment ago—that this is the direction towards which censure or education ought to be directed. Obviously at the present moment Parliament, even were it not engaged on weightier matters, could not embark on penal legislation, for patent facts announce that in such a matter the public loves its liberty, and there are doubtless legislators, even in both Houses, who look outside the orthodox medical ranks. Hence, for our own part, we look for no sudden and dramatic change, and we are inclined to say that members of the profession might neglect the topic of unqualified practice without any sacrifice of their responsibility and without any gain to its popular welcome.

If the subject must be discussed by the profession it were well to have it discussed without exaggeration. It is quite true, as we are often told, that the results may be disastrous to the sufferer, but nothing justifies a tone which suggests that this result arrives frequently. The sustained habit of the public rests upon the experience that ordinary sore throat does not usually prove to be diphtheria, and that there are many stomach-aches which do not find appendicitis as their interpretation. For any individual attacked with such symptoms there is the risk of the graver malady, but numerically the chances are in his favour. The really prudent man will avoid the risk, but few, or none, will be driven to this selection by an exaggeration of the risk incidence. A contradiction of this form of statement is provided by daily experience, and on this the conclusion is apt to be reached that in a practical sense there is no danger at all. Over-statement both spoils a good case and nullifies good counsel. That a pharmacist or any other person should not pretend to be what he is not, goes without saying, and conduct of this order would be allowed by all to be "immoral." But the adjective is surely excessive if applied to the action of a person who on request supplies something which gives relief to a person who on his own responsibility concludes that his discomforts are slight and without risk. That neither of the two persons concerned is acting wisely we may agree, but we had better abstain from too exalted a censure.

